# Brain Herniation Secondary to Cerebrospinal Fluid Leak Following Elective Lumbar Spine Surgery

**DOI:** 10.7759/cureus.20266

**Published:** 2021-12-08

**Authors:** Andrew Zhang, Junho Song, John K Czerwein

**Affiliations:** 1 Orthopaedic Surgery, Louisiana State University Health Sciences Center, Shreveport, USA; 2 Orthopaedic Surgery, Hospital for Special Surgery, New York, USA; 3 Orthopaedic Surgery, Warren Alpert Medical School of Brown University, Providence, USA

**Keywords:** complication, elective, lumbar, transtentorial herniation, spine surgery, brain herniation, dural leak, csf leak

## Abstract

Lumbar spine surgery can be complicated by perioperative cerebrospinal fluid (CSF) leak. However, development of brain herniation secondary to CSF leak following lumbar spine surgery has not been previously reported in the current literature. This case report describes a 48-year-old woman who, after a revision lumbar decompression and fusion, experienced CSF leak followed by development of brain herniation, which resulted in patient demise. The postoperative period was complicated by patient nonadherence to conservative management of CSF leak.

## Introduction

A cerebrospinal fluid (CSF) leak can arise from various etiologies, including trauma, hydrocephalus, infection, iatrogenic, and spontaneous [1,2]. Unfortunately, it is one of the more commonly reported complications associated with lumbar spine surgery [3,4]. Untreated or inadequately managed CSF leaks have been associated with a wide variety of complications, such as intracranial hypotension, meningitis, coma, worsening back pain, arachnoiditis, and general poor functional outcome [1,3,5]. Here, we describe the rare case of a patient developing a brain herniation following a CSF leak created from an elective spine surgery that has never been previously reported in the literature.

## Case presentation

**Figure 1:**
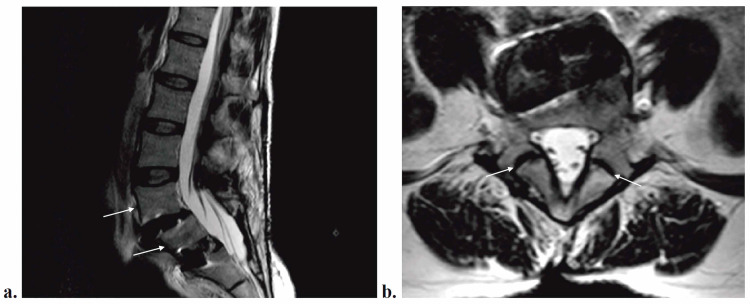
MRI of Lumbar Spine Sagittal (a) and axial (b) MRI of the lumbar spine, illustrating grade 1 anterolisthesis of L4 on L5 and L5 on S1 and bilateral pars defects of L5. Interbody devices from a previous surgery are present at L4-5 and L5-S1 disc spaces. MRI, magnetic resonance imaging.

A 48-year-old female with a history of multiple previous spine surgeries presented with continued complaints of back and right lower extremity radicular pain. Physical examination was remarkable for tenderness to palpation along the lower lumbar spine but otherwise was neurologically intact without obvious provocative maneuvers. A magnetic resonance imaging (MRI) was obtained showing significant scar tissue but otherwise stable laminectomies at L4-5 and L5-S1 with known anterior interbody at the L5-S1 level. There was evidence of anterolisthesis at L4-5 due to bilateral pars defects of L5, as well as an anterolisthesis at L5-S1 (Figure 1). With concern for pseudoarthrosis and radiculopathy secondary to extensive scar tissue formation, she was indicated for a revision L4-S1 decompression and fusion with instrumentation.

A small, iatrogenic dorsal CSF leak was noted toward the beginning of the surgery. The leak was repaired with a simple Prolene suture, and there was no evidence of any ongoing leak throughout the remainder of the surgery and confirmed with multiple Valsalva maneuvers. A Hemovac drain was placed, a multiple-layered closure was achieved, and the patient was taken to the recovery room in stable condition.

Because of the dural leak, the patient was advised to remain supine to protect the repair. However, the nursing staff reported on multiple occasions that the patient had been out of bed and walking despite these recommendations. Additionally, the patient was noted to have edible marijuana and an e-cigarette in her possession at the hospital. While she was hospitalized, the patient never experienced vital sign instability, fevers, chills, or night sweats. On postoperative day 2, the Hemovac drain was removed. At the time, the patient complained of back pain but noted significant improvement in her right leg pain. At this point, she did not complain of any headaches. On postoperative day 3, the patient reported feeling well, and her dressing was dry and intact with minimal discharge. She was again instructed to remain lying flat as she was experiencing postural headaches. 

On postoperative days 4 and 5, mild drainage was noted from the wound and drain site; therefore, the incision and drain sites were oversewn. Over the next day, the patient remained noncompliant with bedrest recommendations and continued to experience headaches when getting up from bed. Again, the patient was reminded to lay flat and was informed that if she continued to have postural headaches on postoperative day 7, we would return to the operating room for repair of a probable ongoing dural leak. 

**Figure 2:**
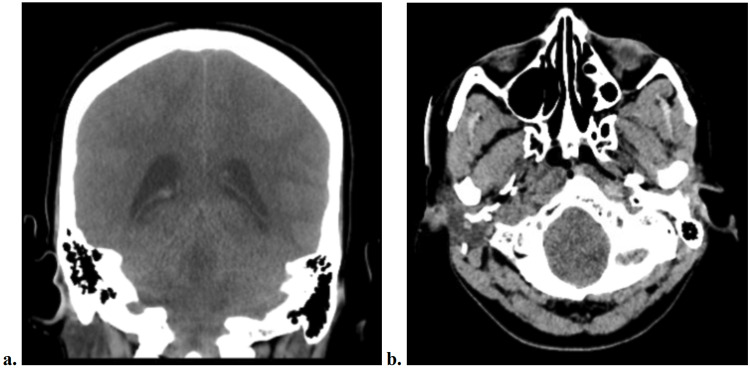
CT Brain Coronal (a) and axial (b) CT brain, illustrating global cerebral edema with effacement of the basilar and quadrigeminal cisterns concerning for transtentorial herniation. CT, computed tomography.

The patient had ongoing headaches and back pain requiring pain medication. On the evening of postoperative day 6, her dressing was noted to be saturated. On postoperative day 7, the patient was noted to be was confused earlier in the day and later found to be unresponsive and unarousable. A code and immediate medicine consult were called, and STAT computed tomography (CT) scan of head was obtained. At this time, the CT scan was interpreted as normal. The patient was then intubated and later transferred to a tertiary care hospital, where the CT scan was again reviewed by another provider and interpreted as a brain herniation (Figure 2). The patient would then expire shortly thereafter.

## Discussion

Procedure-related complications following lumbar spine surgery have been widely reported in the literature, including wound infection, neurologic deficit, hardware malposition, durotomy, urinary tract infection, and deep venous thrombosis [6,7]. Although CSF leaks may occur during lumbar spine surgery [8], brain herniation from this complication has yet to be reported.

Various incidence rates of CSF leak following lumbar spine surgery have been reported, ranging from 5.5% to 21%, with higher rates observed for revision surgeries compared to primary surgeries [1]. Clinically significant CSF leaks can present with severe, persistent headaches, which worsen with upright positioning, neck stiffness, nausea, diplopia, tinnitus, photophobia, and blurred vision [1,9]. Current literature on complications associated with CSF leaks include intracranial hypotension, meningitis, coma, worsening back pain, arachnoiditis, and general poor functional outcome [1,3,5]. Other rare complications have also been reported, including intracranial subdural hematoma, spinal cord herniation, pneumocephalus, pneumorachis, and posterior reversible encephalopathy syndrome [3,10,11]. 

Another rare complication associated with CSF leaks is foramen magnum syndrome, a constellation of neurologic findings in the setting of cerebellar tonsillar ectopia. Badve et al. present a series of two cases of foramen magnum syndrome seen after iatrogenic CSF leaks during lumbar spine surgery [12]. In these cases, patients experienced CSF leaks following decompressive laminectomy and subsequently developed pathology within the foramen magnum, including tonsillar ectopia, posterior fossa hemorrhage, and hydrocephalus. This was likely secondary to distal CSF diversion producing intracranial hypotension, resulting in a negative pressure gradient between the cranial and spinal theca, which draws the tonsils through the foramen magnum. 

Given the possibility of these fatal complications, early detection and management of postoperative CSF leaks are crucial. Management of a patient with CSF leak after spinal surgery may involve conservative measures such as bedrest, analgesics, and hydration or require surgery, including primary repair or epidural patching [13,14]. This patient was initially recommended bedrest to aid in reinforcement of the primary repair of the dural tear. However, the patient was noted to be noncompliant on multiple occasions. With continued drainage and the onset of postural headaches, the plan was to return to the operating room for possible dural repair. 

The possible mechanism of the brain herniation in the present case may be related to changes in intracranial pressure secondary to CSF leak. Intracranial hypotension typically results from loss of cerebrospinal fluid, and CSF leak is the prevailing theory for the etiology of spontaneous intracranial hypotension [15-17]. Significant changes in intracranial pressure due to changes in CSF volume can result in brain herniation. Kim et al. describe a case in which CSF drainage for ruptured aneurysm resulted in a phenomenon termed “brain sag.” Brain sag refers to herniation of the brain stem caused by the loss of significant CSF, provoking intracranial hypotension. This may present as altered mental status, pupillary asymmetry, and decerebrate posturing. In these cases, placing the patient in Trendelenburg position can rapidly improve neurological status [18]. Wicklund et al. report a patient with confirmed CSF leak who developed frontotemporal brain sagging syndrome, characterized by downward displacement of cerebellar tonsils and midbrain swelling on MRI. This condition can clinically mimic behavioral variant frontotemporal dementia, producing changes in behavior and executive functions accompanied by a headache [19]. In our patient, it is possible that an ongoing CSF leak following lumbar spine surgery led to significant intracranial hypotension, ultimately resulting in brain herniation. Similar to the mechanism of foramen magnum syndrome, significant acute intracranial hypotension would produce a negative pressure gradient, leading to diffusion of the brain parenchymal tissue.

Alternatively, the patient may have had an underlying congenital abnormality which predisposed her to developing a brain herniation. Chiari malformations are characterized by herniation of the cerebellar vermis and/or tonsils. However, this possibility could not be thoroughly explored in the present case as post-mortem MRI would be necessary. Furthermore, while late presentations are possible, congenital malformations would be more likely to present with symptoms at an earlier age. The incidence of asymptomatic Chiari malformation is reported to be about 2% [20].

## Conclusions

We illustrate the case of a CSF leak and consequential brain herniation in a patient following elective lumbar spine surgery. These events arose despite the intraoperative identification and repair of the CSF leak. Postoperatively, the patient developed postural headaches and did not adhere to the repeated recommendations to remain supine. The patient ultimately expired one week after the surgery. CT scan of the head revealed transtentorial brain herniation.

This represents the first described case in the literature of a brain herniation secondary to CSF leak following spine surgery. Spine surgeons should be aware of this rare, but potentially life-threatening complication, and patients should be appropriately counseled, especially in the setting of patient noncompliance. 
